# Supplementary material for: Pyruvate Dehydrogenase Kinase 1 inhibition mediated oxidative phosphorylation enhancement in cartilage promotes osteoarthritis progression
Source: BMC Musculoskelet Disord. 2023 Jul 20;24:597. doi: 10.1186/s12891-023-06585-6 (PMC10357736; doi:10.1186/s12891-023-06585-6)
Supplement: Supplementary file 1 — Additional file 1. [file 12891_2023_6585_MOESM1_ESM.zip › KEGG.Gsea.1653622432464/KEGG_ALZHEIMERS_DISEASE.html]

Details for gene set KEGG\_ALZHEIMERS\_DISEASE[GSEA]

|  || Dataset | 55457\_collapsed\_to\_symbols.cls.cls #OA\_versus\_normal.cls.cls #OA\_versus\_normal\_repos |
| Phenotype | cls.cls#OA\_versus\_normal\_repos |
| Upregulated in class | OA |
| GeneSet | KEGG\_ALZHEIMERS\_DISEASE |
| Enrichment Score (ES) | 0.41058877 |
| Normalized Enrichment Score (NES) | 2.0205495 |
| Nominal p-value | 0.0 |
| FDR q-value | 0.0021446894 |
| FWER p-Value | 0.012 |
Table: GSEA Results Summary

  

Fig 1: Enrichment plot: KEGG\_ALZHEIMERS\_DISEASE      
 Profile of the Running ES Score & Positions of GeneSet Members on the Rank Ordered List

  

| SYMBOL | TITLE | RANK IN GENE LIST | RANK METRIC SCORE | RUNNING ES | CORE ENRICHMENT || 1 | UQCR10 | "ubiquinol-cytochrome c reductase, complex III subunit X [Source:HGNC Symbol;Acc:HGNC:30863]" | 53 | 0.987 | 0.0150 | Yes |
| 2 | NDUFA8 | NADH:ubiquinone oxidoreductase subunit A8 [Source:HGNC Symbol;Acc:HGNC:7692] | 67 | 0.961 | 0.0327 | Yes |
| 3 | NDUFA4L2 | NDUFA4 mitochondrial complex associated like 2 [Source:HGNC Symbol;Acc:HGNC:29836] | 150 | 0.861 | 0.0430 | Yes |
| 4 | NDUFS3 | NADH:ubiquinone oxidoreductase core subunit S3 [Source:HGNC Symbol;Acc:HGNC:7710] | 177 | 0.836 | 0.0573 | Yes |
| 5 | NDUFA1 | NADH:ubiquinone oxidoreductase subunit A1 [Source:HGNC Symbol;Acc:HGNC:7683] | 194 | 0.826 | 0.0721 | Yes |
| 6 | NDUFA10 | NADH:ubiquinone oxidoreductase subunit A10 [Source:HGNC Symbol;Acc:HGNC:7684] | 243 | 0.788 | 0.0836 | Yes |
| 7 | NDUFB6 | NADH:ubiquinone oxidoreductase subunit B6 [Source:HGNC Symbol;Acc:HGNC:7701] | 262 | 0.779 | 0.0974 | Yes |
| 8 | PSEN1 | presenilin 1 [Source:HGNC Symbol;Acc:HGNC:9508] | 425 | 0.695 | 0.0982 | Yes |
| 9 | ATP5MC1 | ATP synthase membrane subunit c locus 1 [Source:HGNC Symbol;Acc:HGNC:841] | 465 | 0.674 | 0.1083 | Yes |
| 10 | UQCRC1 | ubiquinol-cytochrome c reductase core protein 1 [Source:HGNC Symbol;Acc:HGNC:12585] | 473 | 0.672 | 0.1208 | Yes |
| 11 | NDUFB3 | NADH:ubiquinone oxidoreductase subunit B3 [Source:HGNC Symbol;Acc:HGNC:7698] | 483 | 0.667 | 0.1330 | Yes |
| 12 | NDUFS4 | NADH:ubiquinone oxidoreductase subunit S4 [Source:HGNC Symbol;Acc:HGNC:7711] | 531 | 0.647 | 0.1419 | Yes |
| 13 | NDUFA3 | NADH:ubiquinone oxidoreductase subunit A3 [Source:HGNC Symbol;Acc:HGNC:7686] | 550 | 0.641 | 0.1530 | Yes |
| 14 | ATP5PD | ATP synthase peripheral stalk subunit d [Source:HGNC Symbol;Acc:HGNC:845] | 564 | 0.635 | 0.1643 | Yes |
| 15 | COX5B | cytochrome c oxidase subunit 5B [Source:HGNC Symbol;Acc:HGNC:2269] | 585 | 0.628 | 0.1750 | Yes |
| 16 | NDUFA4 | NDUFA4 mitochondrial complex associated [Source:HGNC Symbol;Acc:HGNC:7687] | 624 | 0.613 | 0.1840 | Yes |
| 17 | COX6A1 | cytochrome c oxidase subunit 6A1 [Source:HGNC Symbol;Acc:HGNC:2277] | 653 | 0.605 | 0.1935 | Yes |
| 18 | NCSTN | nicastrin [Source:HGNC Symbol;Acc:HGNC:17091] | 666 | 0.601 | 0.2043 | Yes |
| 19 | MAPK3 | mitogen-activated protein kinase 3 [Source:HGNC Symbol;Acc:HGNC:6877] | 757 | 0.579 | 0.2085 | Yes |
| 20 | ATP5PO | ATP synthase peripheral stalk subunit OSCP [Source:HGNC Symbol;Acc:HGNC:850] | 840 | 0.558 | 0.2129 | Yes |
| 21 | NDUFA2 | NADH:ubiquinone oxidoreductase subunit A2 [Source:HGNC Symbol;Acc:HGNC:7685] | 878 | 0.549 | 0.2207 | Yes |
| 22 | NDUFB5 | NADH:ubiquinone oxidoreductase subunit B5 [Source:HGNC Symbol;Acc:HGNC:7700] | 902 | 0.542 | 0.2294 | Yes |
| 23 | FADD | Fas associated via death domain [Source:HGNC Symbol;Acc:HGNC:3573] | 924 | 0.534 | 0.2382 | Yes |
| 24 | NDUFC2 | NADH:ubiquinone oxidoreductase subunit C2 [Source:HGNC Symbol;Acc:HGNC:7706] | 929 | 0.532 | 0.2482 | Yes |
| 25 | NDUFA9 | NADH:ubiquinone oxidoreductase subunit A9 [Source:HGNC Symbol;Acc:HGNC:7693] | 943 | 0.529 | 0.2575 | Yes |
| 26 | NDUFB4 | NADH:ubiquinone oxidoreductase subunit B4 [Source:HGNC Symbol;Acc:HGNC:7699] | 968 | 0.525 | 0.2658 | Yes |
| 27 | UQCRQ | ubiquinol-cytochrome c reductase complex III subunit VII [Source:HGNC Symbol;Acc:HGNC:29594] | 986 | 0.521 | 0.2746 | Yes |
| 28 | NDUFB7 | NADH:ubiquinone oxidoreductase subunit B7 [Source:HGNC Symbol;Acc:HGNC:7702] | 1033 | 0.510 | 0.2810 | Yes |
| 29 | CALM3 | calmodulin 3 [Source:HGNC Symbol;Acc:HGNC:1449] | 1039 | 0.510 | 0.2905 | Yes |
| 30 | GRIN2A | glutamate ionotropic receptor NMDA type subunit 2A [Source:HGNC Symbol;Acc:HGNC:4585] | 1054 | 0.506 | 0.2992 | Yes |
| 31 | NDUFB1 | NADH:ubiquinone oxidoreductase subunit B1 [Source:HGNC Symbol;Acc:HGNC:7695] | 1055 | 0.506 | 0.3091 | Yes |
| 32 | NDUFS7 | NADH:ubiquinone oxidoreductase core subunit S7 [Source:HGNC Symbol;Acc:HGNC:7714] | 1071 | 0.501 | 0.3176 | Yes |
| 33 | NDUFC1 | NADH:ubiquinone oxidoreductase subunit C1 [Source:HGNC Symbol;Acc:HGNC:7705] | 1091 | 0.499 | 0.3259 | Yes |
| 34 | NDUFA7 | NADH:ubiquinone oxidoreductase subunit A7 [Source:HGNC Symbol;Acc:HGNC:7691] | 1097 | 0.496 | 0.3351 | Yes |
| 35 | COX8A | cytochrome c oxidase subunit 8A [Source:HGNC Symbol;Acc:HGNC:2294] | 1105 | 0.494 | 0.3442 | Yes |
| 36 | NDUFS8 | NADH:ubiquinone oxidoreductase core subunit S8 [Source:HGNC Symbol;Acc:HGNC:7715] | 1208 | 0.474 | 0.3454 | Yes |
| 37 | NDUFS1 | NADH:ubiquinone oxidoreductase core subunit S1 [Source:HGNC Symbol;Acc:HGNC:7707] | 1229 | 0.471 | 0.3530 | Yes |
| 38 | CALML5 | calmodulin like 5 [Source:HGNC Symbol;Acc:HGNC:18180] | 1261 | 0.465 | 0.3596 | Yes |
| 39 | NAE1 | NEDD8 activating enzyme E1 subunit 1 [Source:HGNC Symbol;Acc:HGNC:621] | 1276 | 0.463 | 0.3675 | Yes |
| 40 | ATP5PB | ATP synthase peripheral stalk-membrane subunit b [Source:HGNC Symbol;Acc:HGNC:840] | 1293 | 0.459 | 0.3752 | Yes |
| 41 | NDUFS6 | NADH:ubiquinone oxidoreductase subunit S6 [Source:HGNC Symbol;Acc:HGNC:7713] | 1417 | 0.441 | 0.3741 | Yes |
| 42 | PSENEN | "presenilin enhancer, gamma-secretase subunit [Source:HGNC Symbol;Acc:HGNC:30100]" | 1529 | 0.424 | 0.3737 | Yes |
| 43 | NDUFV2 | NADH:ubiquinone oxidoreductase core subunit V2 [Source:HGNC Symbol;Acc:HGNC:7717] | 1642 | 0.407 | 0.3728 | Yes |
| 44 | NDUFV1 | NADH:ubiquinone oxidoreductase core subunit V1 [Source:HGNC Symbol;Acc:HGNC:7716] | 1663 | 0.404 | 0.3791 | Yes |
| 45 | SNCA | synuclein alpha [Source:HGNC Symbol;Acc:HGNC:11138] | 1800 | 0.384 | 0.3759 | Yes |
| 46 | SDHB | succinate dehydrogenase complex iron sulfur subunit B [Source:HGNC Symbol;Acc:HGNC:10681] | 1818 | 0.382 | 0.3820 | Yes |
| 47 | COX7A1 | cytochrome c oxidase subunit 7A1 [Source:HGNC Symbol;Acc:HGNC:2287] | 2045 | 0.356 | 0.3712 | Yes |
| 48 | TNF | tumor necrosis factor [Source:HGNC Symbol;Acc:HGNC:11892] | 2098 | 0.350 | 0.3740 | Yes |
| 49 | COX4I1 | cytochrome c oxidase subunit 4I1 [Source:HGNC Symbol;Acc:HGNC:2265] | 2167 | 0.342 | 0.3753 | Yes |
| 50 | ATP5F1C | ATP synthase F1 subunit gamma [Source:HGNC Symbol;Acc:HGNC:833] | 2190 | 0.339 | 0.3801 | Yes |
| 51 | PLCB4 | phospholipase C beta 4 [Source:HGNC Symbol;Acc:HGNC:9059] | 2217 | 0.337 | 0.3847 | Yes |
| 52 | SDHA | succinate dehydrogenase complex flavoprotein subunit A [Source:HGNC Symbol;Acc:HGNC:10680] | 2231 | 0.335 | 0.3902 | Yes |
| 53 | NDUFS5 | NADH:ubiquinone oxidoreductase subunit S5 [Source:HGNC Symbol;Acc:HGNC:7712] | 2267 | 0.330 | 0.3938 | Yes |
| 54 | ATP5MC2 | ATP synthase membrane subunit c locus 2 [Source:HGNC Symbol;Acc:HGNC:842] | 2371 | 0.318 | 0.3920 | Yes |
| 55 | NDUFB2 | NADH:ubiquinone oxidoreductase subunit B2 [Source:HGNC Symbol;Acc:HGNC:7697] | 2460 | 0.307 | 0.3910 | Yes |
| 56 | COX7A2L | cytochrome c oxidase subunit 7A2 like [Source:HGNC Symbol;Acc:HGNC:2289] | 2517 | 0.302 | 0.3925 | Yes |
| 57 | BACE1 | beta-secretase 1 [Source:HGNC Symbol;Acc:HGNC:933] | 2597 | 0.291 | 0.3920 | Yes |
| 58 | NDUFB8 | NADH:ubiquinone oxidoreductase subunit B8 [Source:HGNC Symbol;Acc:HGNC:7703] | 2623 | 0.288 | 0.3956 | Yes |
| 59 | ATP5MC3 | ATP synthase membrane subunit c locus 3 [Source:HGNC Symbol;Acc:HGNC:843] | 2643 | 0.286 | 0.3997 | Yes |
| 60 | ATP5F1D | ATP synthase F1 subunit delta [Source:HGNC Symbol;Acc:HGNC:837] | 2701 | 0.279 | 0.4006 | Yes |
| 61 | CACNA1F | calcium voltage-gated channel subunit alpha1 F [Source:HGNC Symbol;Acc:HGNC:1393] | 2726 | 0.278 | 0.4042 | Yes |
| 62 | APOE | apolipoprotein E [Source:HGNC Symbol;Acc:HGNC:613] | 2776 | 0.272 | 0.4056 | Yes |
| 63 | ATP2A1 | ATPase sarcoplasmic/endoplasmic reticulum Ca2+ transporting 1 [Source:HGNC Symbol;Acc:HGNC:811] | 2781 | 0.272 | 0.4106 | Yes |
| 64 | COX6B1 | cytochrome c oxidase subunit 6B1 [Source:HGNC Symbol;Acc:HGNC:2280] | 2915 | 0.259 | 0.4052 | No |
| 65 | ATP5F1B | ATP synthase F1 subunit beta [Source:HGNC Symbol;Acc:HGNC:830] | 3024 | 0.247 | 0.4015 | No |
| 66 | NDUFS2 | NADH:ubiquinone oxidoreductase core subunit S2 [Source:HGNC Symbol;Acc:HGNC:7708] | 3072 | 0.243 | 0.4026 | No |
| 67 | SDHC | succinate dehydrogenase complex subunit C [Source:HGNC Symbol;Acc:HGNC:10682] | 3145 | 0.235 | 0.4015 | No |
| 68 | COX7A2 | cytochrome c oxidase subunit 7A2 [Source:HGNC Symbol;Acc:HGNC:2288] | 3164 | 0.234 | 0.4047 | No |
| 69 | CASP3 | caspase 3 [Source:HGNC Symbol;Acc:HGNC:1504] | 3202 | 0.230 | 0.4062 | No |
| 70 | CDK5 | cyclin dependent kinase 5 [Source:HGNC Symbol;Acc:HGNC:1774] | 3400 | 0.211 | 0.3949 | No |
| 71 | COX7B | cytochrome c oxidase subunit 7B [Source:HGNC Symbol;Acc:HGNC:2291] | 3452 | 0.207 | 0.3949 | No |
| 72 | PLCB1 | phospholipase C beta 1 [Source:HGNC Symbol;Acc:HGNC:15917] | 3724 | 0.183 | 0.3772 | No |
| 73 | BAD | BCL2 associated agonist of cell death [Source:HGNC Symbol;Acc:HGNC:936] | 3788 | 0.178 | 0.3758 | No |
| 74 | PPP3CA | protein phosphatase 3 catalytic subunit alpha [Source:HGNC Symbol;Acc:HGNC:9314] | 3803 | 0.176 | 0.3781 | No |
| 75 | UQCRH | ubiquinol-cytochrome c reductase hinge protein [Source:HGNC Symbol;Acc:HGNC:12590] | 3827 | 0.174 | 0.3797 | No |
| 76 | FAS | Fas cell surface death receptor [Source:HGNC Symbol;Acc:HGNC:11920] | 3828 | 0.173 | 0.3830 | No |
| 77 | NDUFA6 | NADH:ubiquinone oxidoreductase subunit A6 [Source:HGNC Symbol;Acc:HGNC:7690] | 3834 | 0.173 | 0.3860 | No |
| 78 | MAPT | microtubule associated protein tau [Source:HGNC Symbol;Acc:HGNC:6893] | 3976 | 0.158 | 0.3780 | No |
| 79 | UQCRC2 | ubiquinol-cytochrome c reductase core protein 2 [Source:HGNC Symbol;Acc:HGNC:12586] | 4020 | 0.154 | 0.3776 | No |
| 80 | CACNA1S | calcium voltage-gated channel subunit alpha1 S [Source:HGNC Symbol;Acc:HGNC:1397] | 4031 | 0.153 | 0.3798 | No |
| 81 | COX6C | cytochrome c oxidase subunit 6C [Source:HGNC Symbol;Acc:HGNC:2285] | 4036 | 0.152 | 0.3825 | No |
| 82 | SDHD | succinate dehydrogenase complex subunit D [Source:HGNC Symbol;Acc:HGNC:10683] | 4055 | 0.151 | 0.3840 | No |
| 83 | HSD17B10 | hydroxysteroid 17-beta dehydrogenase 10 [Source:HGNC Symbol;Acc:HGNC:4800] | 4060 | 0.150 | 0.3866 | No |
| 84 | ATP5F1A | ATP synthase F1 subunit alpha [Source:HGNC Symbol;Acc:HGNC:823] | 4137 | 0.143 | 0.3834 | No |
| 85 | ITPR2 | "inositol 1,4,5-trisphosphate receptor type 2 [Source:HGNC Symbol;Acc:HGNC:6181]" | 4222 | 0.135 | 0.3795 | No |
| 86 | NOS1 | nitric oxide synthase 1 [Source:HGNC Symbol;Acc:HGNC:7872] | 4228 | 0.134 | 0.3817 | No |
| 87 | APH1A | "aph-1 homolog A, gamma-secretase subunit [Source:HGNC Symbol;Acc:HGNC:29509]" | 4256 | 0.132 | 0.3821 | No |
| 88 | NDUFAB1 | NADH:ubiquinone oxidoreductase subunit AB1 [Source:HGNC Symbol;Acc:HGNC:7694] | 4445 | 0.118 | 0.3697 | No |
| 89 | COX7C | cytochrome c oxidase subunit 7C [Source:HGNC Symbol;Acc:HGNC:2292] | 4539 | 0.110 | 0.3645 | No |
| 90 | PSEN2 | presenilin 2 [Source:HGNC Symbol;Acc:HGNC:9509] | 4544 | 0.109 | 0.3663 | No |
| 91 | CYC1 | cytochrome c1 [Source:HGNC Symbol;Acc:HGNC:2579] | 4879 | 0.082 | 0.3418 | No |
| 92 | UQCR11 | "ubiquinol-cytochrome c reductase, complex III subunit XI [Source:HGNC Symbol;Acc:HGNC:30862]" | 4917 | 0.078 | 0.3404 | No |
| 93 | UQCRB | ubiquinol-cytochrome c reductase binding protein [Source:HGNC Symbol;Acc:HGNC:12582] | 5003 | 0.073 | 0.3351 | No |
| 94 | COX6A2 | cytochrome c oxidase subunit 6A2 [Source:HGNC Symbol;Acc:HGNC:2279] | 5242 | 0.053 | 0.3175 | No |
| 95 | PPP3CB | protein phosphatase 3 catalytic subunit beta [Source:HGNC Symbol;Acc:HGNC:9315] | 5311 | 0.046 | 0.3131 | No |
| 96 | COX5A | cytochrome c oxidase subunit 5A [Source:HGNC Symbol;Acc:HGNC:2267] | 5329 | 0.045 | 0.3126 | No |
| 97 | IDE | insulin degrading enzyme [Source:HGNC Symbol;Acc:HGNC:5381] | 5491 | 0.033 | 0.3006 | No |
| 98 | UQCRFS1 | "ubiquinol-cytochrome c reductase, Rieske iron-sulfur polypeptide 1 [Source:HGNC Symbol;Acc:HGNC:12587]" | 5509 | 0.032 | 0.2999 | No |
| 99 | ERN1 | endoplasmic reticulum to nucleus signaling 1 [Source:HGNC Symbol;Acc:HGNC:3449] | 5658 | 0.019 | 0.2887 | No |
| 100 | BID | BH3 interacting domain death agonist [Source:HGNC Symbol;Acc:HGNC:1050] | 5715 | 0.014 | 0.2846 | No |
| 101 | ATP5PF | ATP synthase peripheral stalk subunit F6 [Source:HGNC Symbol;Acc:HGNC:847] | 6109 | -0.013 | 0.2540 | No |
| 102 | CALML3 | calmodulin like 3 [Source:HGNC Symbol;Acc:HGNC:1452] | 6348 | -0.032 | 0.2360 | No |
| 103 | CACNA1C | calcium voltage-gated channel subunit alpha1 C [Source:HGNC Symbol;Acc:HGNC:1390] | 6367 | -0.033 | 0.2352 | No |
| 104 | ATP5F1E | ATP synthase F1 subunit epsilon [Source:HGNC Symbol;Acc:HGNC:838] | 6405 | -0.036 | 0.2330 | No |
| 105 | ATP2A3 | ATPase sarcoplasmic/endoplasmic reticulum Ca2+ transporting 3 [Source:HGNC Symbol;Acc:HGNC:813] | 6559 | -0.046 | 0.2219 | No |
| 106 | ITPR1 | "inositol 1,4,5-trisphosphate receptor type 1 [Source:HGNC Symbol;Acc:HGNC:6180]" | 6752 | -0.059 | 0.2080 | No |
| 107 | NDUFA5 | NADH:ubiquinone oxidoreductase subunit A5 [Source:HGNC Symbol;Acc:HGNC:7688] | 6789 | -0.062 | 0.2064 | No |
| 108 | MAPK1 | mitogen-activated protein kinase 1 [Source:HGNC Symbol;Acc:HGNC:6871] | 6853 | -0.068 | 0.2028 | No |
| 109 | PPP3CC | protein phosphatase 3 catalytic subunit gamma [Source:HGNC Symbol;Acc:HGNC:9316] | 7031 | -0.082 | 0.1905 | No |
| 110 | PLCB2 | phospholipase C beta 2 [Source:HGNC Symbol;Acc:HGNC:9055] | 7052 | -0.084 | 0.1905 | No |
| 111 | PLCB3 | phospholipase C beta 3 [Source:HGNC Symbol;Acc:HGNC:9056] | 7118 | -0.090 | 0.1872 | No |
| 112 | GNAQ | G protein subunit alpha q [Source:HGNC Symbol;Acc:HGNC:4390] | 7518 | -0.120 | 0.1582 | No |
| 113 | APP | amyloid beta precursor protein [Source:HGNC Symbol;Acc:HGNC:620] | 7835 | -0.143 | 0.1362 | No |
| 114 | CAPN2 | calpain 2 [Source:HGNC Symbol;Acc:HGNC:1479] | 8114 | -0.164 | 0.1176 | No |
| 115 | CASP9 | caspase 9 [Source:HGNC Symbol;Acc:HGNC:1511] | 8142 | -0.166 | 0.1187 | No |
| 116 | TNFRSF1A | TNF receptor superfamily member 1A [Source:HGNC Symbol;Acc:HGNC:11916] | 8196 | -0.171 | 0.1179 | No |
| 117 | LPL | lipoprotein lipase [Source:HGNC Symbol;Acc:HGNC:6677] | 8374 | -0.184 | 0.1076 | No |
| 118 | CYCS | "cytochrome c, somatic [Source:HGNC Symbol;Acc:HGNC:19986]" | 8410 | -0.186 | 0.1085 | No |
| 119 | CAPN1 | calpain 1 [Source:HGNC Symbol;Acc:HGNC:1476] | 8500 | -0.194 | 0.1053 | No |
| 120 | CHP2 | calcineurin like EF-hand protein 2 [Source:HGNC Symbol;Acc:HGNC:24927] | 8573 | -0.199 | 0.1035 | No |
| 121 | ATF6 | activating transcription factor 6 [Source:HGNC Symbol;Acc:HGNC:791] | 8680 | -0.208 | 0.0992 | No |
| 122 | ADAM10 | ADAM metallopeptidase domain 10 [Source:HGNC Symbol;Acc:HGNC:188] | 8875 | -0.223 | 0.0884 | No |
| 123 | ATP2A2 | ATPase sarcoplasmic/endoplasmic reticulum Ca2+ transporting 2 [Source:HGNC Symbol;Acc:HGNC:812] | 9015 | -0.233 | 0.0820 | No |
| 124 | CASP7 | caspase 7 [Source:HGNC Symbol;Acc:HGNC:1508] | 9232 | -0.251 | 0.0699 | No |
| 125 | MME | membrane metalloendopeptidase [Source:HGNC Symbol;Acc:HGNC:7154] | 9287 | -0.255 | 0.0707 | No |
| 126 | GRIN2C | glutamate ionotropic receptor NMDA type subunit 2C [Source:HGNC Symbol;Acc:HGNC:4587] | 9515 | -0.275 | 0.0582 | No |
| 127 | LRP1 | LDL receptor related protein 1 [Source:HGNC Symbol;Acc:HGNC:6692] | 9540 | -0.277 | 0.0617 | No |
| 128 | BACE2 | beta-secretase 2 [Source:HGNC Symbol;Acc:HGNC:934] | 9838 | -0.305 | 0.0444 | No |
| 129 | CACNA1D | calcium voltage-gated channel subunit alpha1 D [Source:HGNC Symbol;Acc:HGNC:1391] | 9867 | -0.308 | 0.0482 | No |
| 130 | EIF2AK3 | eukaryotic translation initiation factor 2 alpha kinase 3 [Source:HGNC Symbol;Acc:HGNC:3255] | 10025 | -0.324 | 0.0421 | No |
| 131 | CALM1 | calmodulin 1 [Source:HGNC Symbol;Acc:HGNC:1442] | 10136 | -0.334 | 0.0400 | No |
| 132 | RYR3 | ryanodine receptor 3 [Source:HGNC Symbol;Acc:HGNC:10485] | 10344 | -0.355 | 0.0307 | No |
| 133 | ITPR3 | "inositol 1,4,5-trisphosphate receptor type 3 [Source:HGNC Symbol;Acc:HGNC:6182]" | 10390 | -0.360 | 0.0342 | No |
| 134 | CASP8 | caspase 8 [Source:HGNC Symbol;Acc:HGNC:1509] | 10442 | -0.366 | 0.0373 | No |
| 135 | CALM2 | calmodulin 2 [Source:HGNC Symbol;Acc:HGNC:1445] | 10579 | -0.382 | 0.0341 | No |
| 136 | CHP1 | calcineurin like EF-hand protein 1 [Source:HGNC Symbol;Acc:HGNC:17433] | 10940 | -0.423 | 0.0141 | No |
| 137 | CDK5R1 | cyclin dependent kinase 5 regulatory subunit 1 [Source:HGNC Symbol;Acc:HGNC:1775] | 10985 | -0.428 | 0.0189 | No |
| 138 | GSK3B | glycogen synthase kinase 3 beta [Source:HGNC Symbol;Acc:HGNC:4617] | 11157 | -0.451 | 0.0143 | No |
| 139 | GAPDH | glyceraldehyde-3-phosphate dehydrogenase [Source:HGNC Symbol;Acc:HGNC:4141] | 11268 | -0.467 | 0.0148 | No |
| 140 | GRIN2D | glutamate ionotropic receptor NMDA type subunit 2D [Source:HGNC Symbol;Acc:HGNC:4588] | 11306 | -0.472 | 0.0210 | No |
| 141 | GRIN2B | glutamate ionotropic receptor NMDA type subunit 2B [Source:HGNC Symbol;Acc:HGNC:4586] | 11487 | -0.500 | 0.0167 | No |
| 142 | ADAM17 | ADAM metallopeptidase domain 17 [Source:HGNC Symbol;Acc:HGNC:195] | 11931 | -0.579 | -0.0068 | No |
| 143 | IL1B | interleukin 1 beta [Source:HGNC Symbol;Acc:HGNC:5992] | 12105 | -0.622 | -0.0083 | No |
| 144 | GRIN1 | glutamate ionotropic receptor NMDA type subunit 1 [Source:HGNC Symbol;Acc:HGNC:4584] | 12603 | -0.799 | -0.0317 | No |
| 145 | PPP3R1 | "protein phosphatase 3 regulatory subunit B, alpha [Source:HGNC Symbol;Acc:HGNC:9317]" | 12653 | -0.829 | -0.0194 | No |
| 146 | APBB1 | amyloid beta precursor protein binding family B member 1 [Source:HGNC Symbol;Acc:HGNC:581] | 12811 | -0.992 | -0.0124 | No |
| 147 | APAF1 | apoptotic peptidase activating factor 1 [Source:HGNC Symbol;Acc:HGNC:576] | 12812 | -0.993 | 0.0069 | No |
Table: GSEA details [plain text format]

  

Fig 2: KEGG\_ALZHEIMERS\_DISEASE      
 Blue-Pink O' Gram in the Space of the Analyzed GeneSet

  

Fig 3: KEGG\_ALZHEIMERS\_DISEASE: Random ES distribution      
 Gene set null distribution of ES for **KEGG\_ALZHEIMERS\_DISEASE**
